# Health risk appraisal in older people 6: factors associated with self-reported poor vision and uptake of eye tests in older people

**DOI:** 10.1186/1471-2296-14-130

**Published:** 2013-09-03

**Authors:** Steve Iliffe, Kalpa Kharicha, Danielle Harari, Cameron Swift, Gerhard Gillmann, Andreas E Stuck

**Affiliations:** 1 Research Department of Primary Care and Population Health, UCL, Royal Free Campus, Rowland Hill Street, London NW3 2PF, UK; 2 Department of Ageing and Health, St. Thomas’ Hospital, London, UK; 3 Clinical Age Research Unit, Kings College London, London, UK; 4 Department of Social and Preventive Medicine, University of Bern, Bern, Switzerland; 5 University Department of Geriatrics, Spital Bern-Ziegler, Inselspital and University of Bern, Bern, Switzerland

**Keywords:** Older people, Vision loss, General practice, Case finding, Educational level

## Abstract

**Background:**

Although free eye testing is available in the UK from a nation-wide network of optometrists, there is evidence of unrecognised, tractable vision loss amongst older people. A recent review identified this unmet need as a priority for further investigation, highlighting the need to understand public perceptions of eye services and barriers to service access and utilisation. This paper aims to identify risk factors for (1) having poor vision and (2) not having had an eyesight check among community-dwelling older people without an established ophthalmological diagnosis.

**Methods:**

Secondary analysis of self-reported data from the ProAge trial. 1792 people without a known ophthalmological diagnosis were recruited from three group practices in London.

**Results:**

Almost two in ten people in this population of older individuals without known ophthalmological diagnoses had self-reported vision loss, and more than a third of them had not had an eye test in the previous twelve months. In this sample, those with limited education, depressed mood, need for help with instrumental and basic activities of daily living (IADLs and BADLs), and subjective memory complaints were at increased risk of fair or poor self-reported vision. Individuals with basic education only were at increased risk for not having had an eye test in the previous 12 months (OR 1.52, 95% CI 1.17-1.98 p=0.002), as were those with no, or only one chronic condition (OR 1.850, 95% CI 1.382-2.477, p<0.001).

**Conclusions:**

Self-reported poor vision in older people without ophthalmological diagnoses is associated with other functional losses, with no or only one chronic condition, and with depression. This pattern of disorders may be the basis for case finding in general practice. Low educational attainment is an independent determinant of not having had eye tests, as well as a factor associated with undiagnosed vision loss. There are other factors, not identified in this study, which determine uptake of eye testing in those with self-reported vision loss. Further exploration is needed to identify these factors and lead towards effective case finding.

## Background

There is compelling evidence of unmet need for eye care amongst older people with undetected vision loss [1]. By the age of 65, 1 in 6 will become blind or partially sighted, and every day around 100 people in the UK start to lose their sight [2]. Between 12 and 50% of older people have undetected visual loss, with higher prevalence amongst women and risk increasing rapidly with age. As the population ages the prevalence of vision loss from a range of eye disorders linked to ageing processes is likely to increase greatly [3].

A substantial proportion of this visual impairment is due to remedial causes such as refractive errors and cataracts. In the Medical Research Council’s study of screening older people refractive errors accounted for 32% of visual impairment in a 75 and over population. Causes of visual loss and eye disease in the rest of the visually impaired sample were: age related macular degeneration (AMD) (53%), cataract (36%), glaucoma (12%), myopic degeneration (4%) and diabetic eye disease (3%) [4]. A north London study of 1547 people aged 65 and over found that 30% were visually impaired and that 72 % of this impairment could potentially be improved by surgery or spectacles [5].

The negative impact of visual impairment on quality of life, activities of daily living [6] and accidents, including falls [7], is also well documented, adding further weight to argument for focusing on prevention, early detection and timely access to treatment in this age group. The UK Vision Strategy [8], launched in April 2008, aims to improve the eye health of the nation by eliminating avoidable sight loss, supporting those with a visual impairment and enhancing the inclusion, participation and independence of blind and partially sighted people.

Whilst these aims are clearly desirable, methods of identifying those with unrecognised visual loss and encouraging them to take up services that will potentially improve their eyesight and quality of life are not well understood. The most recent update of the Cochrane review on screening for asymptomatic visual impairment shows that screening does not lead to improved vision in the older population [9].

It is not yet clear why remediable poor vision is being missed in an advanced primary care system with easily accessible doctors and nurses who can administer simple screening tests, and a widespread network of community optometrists who offer free NHS sight tests to older people. A review of the prevalence of visual impairment by the Royal National Institute for the Blind identified this problem as a priority for further investigation, highlighting issues of public perception and barriers to service access and utilisation [10]. Given the lack of evidence of benefit for population screening, an alternative approach might be to foster case-finding in general practice, targeting vision assessments at individuals at highest risk of having undetected vision loss.

However, we do not know enough about the characteristics of people with un-assessed or untreated vision loss to be able to describe a clinically obvious group for targeted assessment. Although associations have been demonstrated between visual loss and age, wellbeing, functional ability, social networks and economic position in a descriptive study using data from the English longitudinal study on Ageing (ELSA) data [11], no adjustments were made in this study for confounding relationships and factors. Similarly, analysis of data from the Medical Research Council’s study of screening older people [12] which did make some adjustments for co-morbidities and functional ability is still insufficient to describe those with significant visual impairment in adequate detail.

This study is the sixth in a series on health risk appraisal in older people. Its objectives are to describe the characteristics of community dwelling people aged 65 and over who report poor vision but who have no established ophthalmological diagnosis, and to investigate the relative importance of socio-economic status, educational attainment, social relationships, co-morbidities, depressed mood, cognitive impairment and functional ability on self-reported poor vision and uptake of eye testing.

## Methods

We carried out a secondary analysis of the ProAge dataset using data collected in 2002. The ProAge study was a multi-national randomised controlled trial investigating the effect of Health Risk Appraisal for Older persons in 2001–2002. In this paper we report on the analysis of baseline data. Participants were recruited from GP group practices in London. Eligibility criteria were patients aged 65 years and over; who were living at home without evidence of need for human assistance in performing basic activities of daily living; without known dementia or a terminal illness; who were able to speak English; and who fully completed and returned a postal Probability of Recurrent Admissions questionnaire [13], and a consent form. The Probability of Recurrent Admissions questionnaire is a screening instrument used to identify members of older populations who are at risk for using health services heavily in the future, and these individuals were also excluded from the study (because the RCT was focussed on “well” older people). Local research ethics committee approval was obtained from Brent Medical Ethics Committee and King’s College Hospital Research Ethics Committee. A full account of the methodology of the study is available elsewhere [14].

The dataset for this population includes self-assessment of vision, wearing glasses or lenses, (as excellent, good, fair, poor or very poor) and self-reported diagnoses of cataract, glaucoma and irreversible retinal disease. There were also questions about co-morbidities, medication use, health service use and uptake of preventive services (including optician’s eye tests), the experience of pain, depression and memory problems, social networks and risk of social isolation, self-efficacy, smoking and alcohol consumption, functional ability and falls history, hearing, physical activity and nutrition. Social isolation was measured according to the Lubben Social Network Scale [15], developed specifically for use among older adult populations and used widely in both research and clinical settings [16-19]. A scale of nought to thirty on the Lubben instrument captures the extent of social contact with family and friends, and being at risk of social isolation is defined as having a score of less than twelve. Depressed mood was ascertained with the 5-item Mental Health Inventory Screening Test (MHI-5) [20], one of the subscales of the Short Form-36 (SF-36), which asks questions about how the person felt during the past month. A score ≤ 65 indicated a depressed mood. Activities of daily living were measured using Lawton’s Instrumental Activities of Daily Living scale [21], and subjective memory complaints using Riege’s checklist [22].

Demographic data included educational attainment, income, previous employment, ethnicity and current living arrangements. Full details of the instruments used to collect baseline data, and of the trial design, are reported elsewhere [23].

**Figure 1:**
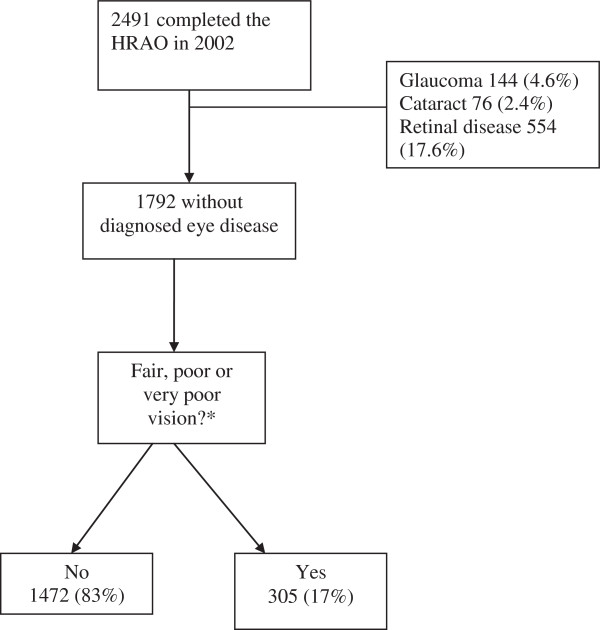
**Flow diagram of study sample derivation for analysis of risk factors for self-reported poor vision.** *Data missing for 15 respondents (0.8%).

The number of self-reported chronic conditions was grouped into 0 or 1, and 2 or more. Because of the tendency to underestimate vision loss [24] , we included those reporting ‘fair’ as well as ‘poor’ or ‘very poor’ vision in the category ‘vision problems’. Those with a diagnosis of glaucoma, retinal disease (of any cause) or cataract were excluded from the study, creating a dataset of those without diagnosed eye disease. The derivation of the sample studied is shown in Figure 1.

Data from the questionnaire was entered on a database designed for the study, with double data entry for purposes of quality control, and analysed using SPSS for Windows (version 15). The characteristics of the study sample were analysed, then the total sample was divided into those with and without self-reported visual problems. In a second step the total sample was divided into those reporting an eye test in the previous twelve months, and those not. Associations between factors previously identified as related to vision loss where analysed using Chi Square tests, and the same factors were analysed for eye testing in the previous twelve months. Two logistic regression analyses were performed using self-reported poor vision and not having had an eye test in the previous twelve months as dependent variables. In addition to age, sex and income, factors which were significantly associated with the dependent variables were included in the regression analyses.

## Results

A total of 2491 people completed the HRAO questionnaire in 2002. After excluding those with known glaucoma (n=144, 4.6%), retinal disease of any cause (n=76, 2.4%) and cataract (n=554, 17.6%), data on visual function was available for 1792 people. Data analysis was performed on this population.

Of the total sample, 944 (52.7%) were women, 531 (29.6%) lived alone, 640 (35.7%) were aged 75 or over, 593 (33.1%) received only the state pension, 1124 (62.7%) reported having only a basic education, and 256 (14.3%) were at risk of social isolation. Two hundred and eighty six (16.0%) had depressed mood, 609 (34.0%) reported need for help with at least one IADL, and 104 (5.8%) with at least one BADL. Two or more chronic conditions were reported by 873 (48.7%) in this population, and 526 (29.9%) used four or more repeat medications. Impaired memory was reported by 173 (9.7%), 840 (46.9%) had changed their activities in the previous 12 months whilst 621 (34.7%) had decreased their activities in the same time period. One thousand and eighty one people (60.3%) reported having an eye test in the previous 12 months.

Three hundred and five people (17.0%) reported fair, poor or very poor vision. Table 1 shows the characteristics of those with self-reported vision problems compared with those without.

**Table 1:** Characteristics of those with and without self-rated poor vision, with single-step logistic regression model

	**No vision problem**	**Poor vision**	**Chi square (df= 1)**		**Adjusted odds ratio**	**95% CI**	**p value**
Characteristic	n (%) 1472	n (%) 305	Chi square	p value			
Female	773 (52.5)	167 (54.8)	0.51	0.48	0.75	0.55 – 1.02	0.07
Aged 75 and over	505 (34.3)	126 (41.3)	5.41	**0.02**	1.02	0.74-1.41	0.90
State pension only	989 (68.3)	169 (56.9)	14.34	**0.001**	0.80	0.58-1.10	0.21
Basic education only*	888 (61.2)	224 (74.9)	20.02	**0.001**	**1.86**	**1.32-2.60**	**<0.001**
Living alone	425 (29.3)	100 (33.3)	1.94	0.16			
At risk of social isolation	195 (13.5)	60 (20.3)	9.27	**0.02**	1.08	0.72-1.64	0.71
Depressed mood	198 (13.5)	85 (34.8)	41.32	**0.001**	**1.55**	**1.06-2.25**	**0.02**
Needs assistance in 1 or more IADLs	442 (31.1)	159 (55.0)	60.25	**0.001**	**1.47**	**1.03-2.09**	**0.03**
Needs assistance in 1 or more BADLs	55 (3.8)	46 (15.4)	61.69	**0.001**	**3.04**	**1.79-5.14**	**<0.001**
Taking 4 or more repeat medications	417 (29.2)	111 (38.9)	10.63	**0.001**	1.08	0.77-1.53	0.66
2 or more chronic conditions	689 (46.8)	184 (60.3)	18.48	**0.001**	1.27	0.90-1.78	0.17
Impaired memory	115 (8.1)	55 (19.2)	33.03	**0.001**	**1.58**	**1.02-2.46**	**0.04**
Recent change in activities	641 (45.3)	189 (65.9)	40.48	**0.001**	1.33	0.89-1.98	0.17
Recent reduction in activities	462 (32.9)	151 (53.5)	43.46	**0.001**	1.30	0.87-1.93	0.20
Eyes tested in the last year	900 (62.2)	174 (58.0)	1.85	0.17			

* Left school before 15.

All the characteristics significantly associated with visual problems were included in a single-step binary logistic regression model, together with age and sex. Table 1 shows the associations between visual problems and characteristics, after adjustment. In the absence of a diagnosis of eye disease, self-reported visual problems were significantly associated with limited education, depressed mood, need for help with instrumental and basic activities of daily living (IADLs and BADLs), and subjective memory complaints.

The characteristics of those who had had a sight test within in the previous 12 months are shown in Table 2. Those who had not had a sight test within the previous 12 months were significantly more likely have limited education and worse health (using the proxies of 4 or more repeat medications and 2 or more chronic conditions for the latter state).

**Table 2:** Characteristics of those with and without eyesight check in last 12 months, with odds ratios for not having an eyesight check

	**No eye-sight check**	**Eye-sight check**	**Chi square (df= 1)**		**Adjusted odds ratio for no eyesight check**	**95% CI**	**p value**
Characteristic	n (%) 681	n (%) 1081	Chi square	p value			
Female	353 (51.8)	569 (52.6)	0.11	0.74	0.86	0.66-1.13	0.28
Aged 75 and over	240 (35.2)	388 (35.9)	0.11	0.78	0.86	0.89-1.16	0.33
State pension only	237 (35.3)	344 (32.3)	1.69	0.19	1.22	0.91-1.63	0.19
Basic education only	466 (69.2)	640 (59.9)	15.47	**<0.001**	**1.52**	**1.17-1.98**	**0.002**
Living alone	208 (31.0)	315 (29.4)	0.47	0.50			
At risk of social isolation	103 (15.4)	150 (14.1)	0.43	0.44			
Depressed mood	103 (15.3)	173 (16.1)	0.21	0.65			
Needs assistance in 1 or more IADLs	224 (33.8)	373 (35.9)	0.73	0.39			
Needs assistance in 1 or more BADLs	44 (6.5)	56 (5.2)	1.23	0.27			
Taking 4 or more repeat medications	169 (25.6)	359 (34.5)	14.63	**<0.001**	0.85	0.61-1.17	0.31
None or only one chronic condition	275 (40.4)	582 (54.1)	31.36	**<0.001**	**1.85**	**1.38-2.48**	**<0.001**
Impaired memory	63 (9.7)	104 (9.9)	0.02	0.89			
Recent change in activities	316 (48.1)	510 (49.2)	0.21	0.65			
Recent reduction in activities	244 (37.5)	366 (35.6)	0.67	0.41			
Fair, poor or very poor vision	74 (16.3)	124 (15.3)	0.19	0.66			

A similar regression analysis was performed (Table 2) using the same characteristics as independent variables, and not having had an eye test in the previous 12 months as the dependent variable. Those with only basic education were significantly more likely not to have had an eye test (OR 1.52 95% CI 1.17-1.98), as were those with no or only one chronic condition (OR 1.85, 95% CI 1.38-2.48, p<0.001). Having two or more chronic conditions reduced the likelihood of not having had an eye test (OR 0.54, 95% CI 0.41-0.72, p<0.001). There was no statistically significant association with other characteristics.

## Discussion

Nearly two in ten people in this population of relatively well older people had self-reported poor vision unrelated to known eye disease and over a third of them had not had an eye test in the previous twelve months. Such vision loss appears to be one component of a wider pattern of disability, and also to be related to lower educational attainment and lower income. Not having had an eye test in the previous twelve months was associated with lower educational attainment. Having two or more chronic conditions was associated with a significantly greater likelihood of having had an eye test.

The depth of information collected from participants in the ProAge trial allows for more complex modelling of factors associated with self-reported vision loss (unrelated to known eye disease) and use of eye screening services than previously carried out. The data is relatively old, but we have seen nothing to suggest a change in patterns of health seeking behaviour for visual symptoms in older people. The original study excluded those with severe disabilities, and we excluded from this analysis those who reported ophthalmological diagnoses, which may mean that we have underestimated the prevalence of undetected vision loss. However the finding that those with two or more chronic conditions were more likely to have eye tests suggests that those with more medical problems either get more services and attention, including assessment of vision, or are more responsive to changes in vision. Recall bias may mean that some people underestimate or overestimate how much time has passed since they had an eye test. In our view the sample is large enough to allow these estimations to cancel each other out. Finally, self-report of poor vision may underestimate the extent of visual impairment, compared with objective eye testing [21].

These findings confirm the association with socioeconomic status found in earlier studies, but only for years in education, not for low income. The proportion having annual eye tests in our sample is greater than in a survey by the Royal National Institute of Blind People of 5,000 people aged 60 and over, which found that almost half (47%) did not have annual eye tests [25]. Our analysis suggests that years in education influences eye testing. However, most of those with low educational attainment have had eye tests. We do not have an explanation for the limited uptake of services by older people with identified visual impairment, even where such services are offered and do provide effective interventions (for example, with uncorrected refraction disorders, cataract and glaucoma). Although some qualitative research has been performed with older people with visual impairments, this has either focused on a particular group like those receiving social care [26] or on a specific need like housing [27].

This study does not suggest that there is an invisible iceberg of undetected vision loss in the older population. Freely available eye testing is used by two thirds of those with or without self-reported vision loss. The number of older people not having eye tests despite having self-reported vision loss is small, making case finding potentially feasible if their characteristics can be described. There is much to learn about the adaptability of older people to impairment, particularly visual impairment [28]. From this study we know that limited education is an independent determinant of not having eye tests, as well as a factor associated with self-reported vision loss. There are likely to be other factors, not identified in this study, which determine uptake of eye testing. Further exploration is needed to identify these factors and lead towards efficient case finding. The accompanying qualitative study describes such factors [29].

## Conclusion

Undetected vision loss is a potentially contributor to disablement, and appears to be related to years in education. Case finding for undetected vision loss could usefully focus on those with depressed mood, functional losses and subjective memory complaints. However, the first step in correcting vision loss is assessment by an optometrist, and there are barriers to this that are not yet fully understood.

## Competing interests

The authors declare that they have no competing interests.

## Authors’ contributions

SI developed the original trial and this secondary analysis, undertook data analysis and drafted this paper; KK developed the original trial and this secondary analysis, undertook data analysis and contributed to this paper; DH developed the original trial and this secondary analysis, and contributed to this paper; CS was PI on the original trial and contributed to this paper; GG lead the statistical analysis the original trial, reviewed this secondary analysis, and contributed to this paper; AS was CI the original trial, reviewed this secondary analysis, and contributed to this paper. All authors read and approved the final manuscript.

## Pre-publication history

The pre-publication history for this paper can be accessed here:

http://www.biomedcentral.com/1471-2296/14/130/prepub
